# What every urologist should know about surgical trials Part I: Are the results valid?

**DOI:** 10.4103/0970-1591.42606

**Published:** 2008

**Authors:** Sohail Bajammal, Mohit Bhandari, Philipp Dahm

**Affiliations:** 1 University of Calgary, Department of Surgery, Calgary, Alberta; 2 McMaster University, Department of Surgery, Hamilton, Ontario, Canada; 3 University of Florida, Department of Urology, Gainesville, Florida, USA

**Keywords:** Bibliographic, databases, evidence-based medicine, information storage and retrieval

## Abstract

Surgical interventions have inherent benefits and associated risks. Before implementing a new therapy, we should ascertain the benefits and risks of the therapy, and assure ourselves that the resources consumed in the intervention will not be exorbitant.

**Materials and Methods:**

We suggest a three-step approach to the critical appraisal of a clinical research study that addresses a question of therapy. Readers should ask themselves the following three questions: Are the study results valid? What are the results? And can I apply them to the care of an individual patient? This first review article on surgical trials will address the question as to whether we consider a study valid or not.

**Results:**

Once the reader has found an article of interest on a urological intervention, it is necessary to assess the quality of the evidence. According to the hierarchy of evidence, a randomized controlled trial is the study design which is the most likely to provide an unbiased estimate of the truth. Important methodological criteria which characterize a high-quality randomized trial include description of allocation concealment, blinding, intention-to-treat analysis, and completeness of follow-up. Failure of investigators to apply these principles may raise concerns about the validity of the study results, thereby making its finding irrelevant.

**Conclusion:**

Assessing the validity of a given study is a critical first step when evaluating a clinical research study. Making this process explicit with guidelines to assess the strength of the available evidence serves to improve patient care. It will also allow urologists to defend therapeutic interventions, based on available evidence and not anecdotes.

## INTRODUCTION

Urologists are faced with daily clinical scenarios that require evidence to support their decision-making. Evidence-based clinical practice (EBCP) has been defined as the “conscientious, explicit and judicious use of current best evidence in making decisions about the care of individual patients.”[1] The quality of evidence depends on the study design; hence, the word “current best” evidence is included in the definition of EBCP. The Oxford Center for Evidence-Based Medicine proposed a hierarchy of evidence for different types of studies, e.g. therapeutic, diagnostic, prognostic, or economic analysis study.[2] In this article, we will discuss therapeutic trials; thus, we will elaborate on the levels of evidence for therapeutic studies. The levels of evidence have been arranged in a pyramid-shaped progression from the highest to the lowest quality of evidence based on the likelihood of systematic error in the study design. The highest level of evidence is the systematic review of randomized trials with homogeneous results. This is followed by high-quality randomized controlled trials (RCTs) with precise results (that is narrow confidence intervals), then low-quality randomized trials and prospective cohort studies. Lower in the pyramid of hierarchy of evidence in therapeutic studies are: retrospective cohort studies, case-control studies, case series and finally expert opinions. Since systematic reviews of randomized trials and high-quality randomized trials represent the highest level of evidence, this article will focus on assessing the methodology of randomized trials with emphasis on the urological literature.

## WHY DO WE NEED RANDOMIZED CONTROLLED TRIALS?

The reason why RCTs are at the top of the hierarchy of evidence is not arbitrary. A high-quality randomized trial avoids the inherent limitations and possible risks of reaching erroneous conclusions potentially derived from observational studies. Case reports and case series are descriptive reports of the intervention and outcome of a single or multiple patients. Since these are uncontrolled reports, it is difficult to make comparisons between different cases series because of differences in the clinical settings of each report. A case-control study is another type of observational study that starts with the patients who already have the outcome of interest; cases are compared with a suitable control group without the outcome. A comparison is then made between the two groups by looking back in time for an intervention or an exposure to certain risk factors. Case-control studies are helpful in studying prognostic factors, especially for rare diseases. However, because case-control studies represent a retrospective study design (i.e., looking back in time), they usually involve examining patients’ charts and asking patients about their remote exposure to risk factors. There is a high likelihood that the charts are not comprehensive and that patients do not recall the information accurately, hence, affecting the credibility of the results of this type of study. A third type of study design is a cohort study. In the cohort study design, a group of people are followed over time to see whether an outcome develops. Ideally, this group meets criteria representative of a population of interest and is followed with well-defined outcomes. Usually, this group is compared with a control group selected on the basis of presence or absence of certain factors. Cohort studies can be prospective, begin at a certain time and followed forward in time, or retrospective, which means that patients’ exposure to certain risk factors is determined from records and patients are followed from the present to the past. As with any retrospective observational study design, retrospective cohort studies lack the control over the criteria for selecting patients as well as the criteria of the outcome measures. A prospective cohort study design, on the other hand, does not have these disadvantages and is the second best design, after randomized trials, to assess a treatment intervention. One of the major disadvantages of the prospective cohort design is the possibility of selection bias, which means favoring one group at the expense of the other, either consciously or subconsciously, by including patients with favorable prognosis in one group. Randomized controlled trials, on the other hand, avoid the risk of selection bias, inherent in cohort studies, by implementing the concepts of randomization, allocation concealment, and blinding to safeguard against these potential biases. We will discuss these concepts in detail in this article.

Labeling a study as a randomized trial is not enough to guarantee its quality. It is important to follow a guideline to appraise an article about surgical therapy to ensure that: 1) The study is valid with sound methodology, 2) The results are accurate and of clinical significance, and 3) The results are generalizable to the clinical situation under question. The purpose of this article is to review the steps of critical appraisal for surgical trials, covering the three aforementioned aspects of trial methodology, to help guide urologists to implement evidence-based clinical practice. A clinical scenario will be used as a practical exercise to illustrate the use of this guideline in the urological literature.

## CLINICAL SCENARIO

You are in the office seeing the last patient of the day, an overall healthy and sexually active 57-year-old Caucasian female that was referred to you for assessment and treatment of pelvic organ prolapse. Her chief complaints are a sensation of vaginal fullness as well as a palpable mass at the introitus. Her past medical history is notable only for three uncomplicated vaginal deliveries as well as a vaginal hysterectomy for myoma. She denies any symptoms of urinary incontinence and has not undergone any prior incontinence or prolapse surgeries. Physical examination demonstrates a well estrogenized vaginal mucosa and confirms the presence of vaginal prolapse to the hymen that you grade as Stage II according to the pelvic-organ prolapse quantification (POP-Q) system. There is little to none urethral hypermobility. Urodynamic evaluation demonstrates no significant detrusor hyperactivity or evidence of stress urinary incontinence.

The patient has undergone a brief pessary trial but finds this incompatible with her active lifestyle. She wishes to explore more definitive surgical treatment options for prolapse. You counsel her that she would likely benefit from an abdominal sacrocolpopexy and review the potential benefits and risks of this procedure with her. She seems happy with the plan, however, expresses concern about developing leakage after surgery. She asks whether she will need any additional procedures at the time of surgery, indicating that two of her friends who underwent prolapse surgery subsequently developed urinary stress incontinence for which they required additional procedures.

You indicate to her that it is not your practice to offer a prophylactic stress incontinence procedure at the time of abdominal sacrocolpopexy but promise to investigate this option further and discuss it with her upon a return visit. She is agreeable to this plan and schedules a follow-up consultation with you in one week's time.

## THE LITERATURE SEARCH

You recall reading a recent article about the CARE (Colpopexy and Urinary Reduction Efforts) trial that addressed the same problem. Since you are not sure of what is the best evidence available to guide you in treating this patient, you decide to search Medline, using the National Library of Medicine's PubMed database. You use the Clinical Queries link in the PubMed and enter “pelvic prolapse AND urinary stress incontinence” and choose the specific search scope. This search yields 22 “hits.” As you review the titles of the articles, two citations attract your attention.[3,4] Both citations address the same trial. Acknowledging that this search strategy is insufficient to identify all randomized trials in this field, you decide to read the article by Brubaker and colleagues titled “Abdominal sacrocolpopexy with Burch colposuspension to reduce urinary stress incontinence”[4] published in 2006 because it appears to address your particular question.

## SUMMARY OF THE APPRAISED ARTICLE

The study by Brubaker and colleagues is a multi-center randomized trial that compared abdominal sacrocolpopexy combined with Burch colposuspension in 157 women with abdominal sacrocolpopexy without colposuspension in 165 women in terms of their urinary stress incontinence and urge symptoms three months after surgery.[4] Women were included if they had Stage II to IV pelvic organ prolapse, had no symptoms of urinary stress incontinence and no contraindications to colposuspension. The data and safety monitoring board of the study recommended stopping the trial after the first interim analysis (when outcomes were available for 232 women) because of the significant difference between the two groups. The planned sample size was 480, but because the trial was stopped early, the total number of recruited women was 322. Three months after surgery, 23.8% of the women in the Burch group and 44.1% of the control group met one or more of the criteria for stress incontinence (P < 0.001). There was no difference between the Burch group and the control group in the frequency of urge incontinence (32.7% vs. 38.4%, *P* = 0.48).

## HOW TO USE AN ARTICLE ABOUT A SURGICAL THERAPY?

Before implementing a new therapy, you should ascertain its benefits and risks and be assured that there is enough evidence to support your decision to embark on the new therapy. In this article, we suggest a three-step approach to critically appraise articles about surgical therapy in the urological literature.[5] First, assess whether the study is valid enough for you to believe its results. In other words, do the results of the study represent an unbiased estimate of the treatment effect, or have they been influenced in a systematic fashion to lead to a false conclusion. Second, review the results and assess how clinically significant they are. Third, assess whether you can extrapolate the results of the study to your clinical problem and whether implementing the new intervention would benefit your patients when considering the benefits and risks of the intervention [Table 1].

**Table 1:** Users’ guide to the urological literature: How to use an article about therapy

**First: Are the results valid?**
Did the two groups begin the study with a similar prognosis? Did the investigators take into account a a learning curve? Were the patients randomized? Was the allocation concealed? Were the patients stratified? Were all patients’ data analyzed in the group to which they were randomized? Were the patients in the two groups similar with respect to konwn prognistic factors? Did the two groups retain a similar prognosis after the study started? Were the patients aware of group allocation? Were the surgeons aware of group allocation? Were the outcome assessors aware of group allocation? Was the follow-up complete?
**Second: What are the results?**
How large was the treatment effect?
How precise was the estimate of the treatment effect?
**Thrid: Can I apply the results to patient care?**
Were the study patients similar to my patient?
Were all clinically important outcomes considered?
Are the likely treatment benefits worth the potential harm and costs?

This list was modified from Users’ Guides to the Medical Literature: A manual for evidence-based clinical practice, by Guyatt G and Rennie D.[5]

### First: Are the results valid?

#### 1. Did the two groups begin the study with a similar prognosis?
Did the investigators take into account the learning curve?

When critically appraising surgical trials, unlike drug trials, it is of paramount importance to pay attention to differential expertise bias.[6–8] Individual surgeons tend to primarily use a single approach, with which they are most experienced and comfortable with, to treat a certain problem. For example, to treat a certain problem, surgeon X has more experience in procedure A than procedure B. On the other hand, surgeon Y has more experience in procedure B. If surgeon X treats 70% of patients in Groups 1 and 2, while surgeon Y treats 30% of patients in both groups, the results of the trial will be biased towards procedure A. Statistically, bias means “any trend in the collection, analysis, interpretation, publication, or review of data that can lead to conclusions that are systematically different from the truth.”[9]

The magnitude of bias that differential expertise can introduce into the study depends on three considerations: 1) Whether the number of participating surgeons with expertise in both procedures is equal in both groups. 2) How steep the learning curve of the new procedure is. In other words, how many procedures need to be performed by the surgeon in order to be eligible to enroll patients in the trials? 3) Whether the comparison group is undergoing a “new” technique that is technically challenging. In this situation, the results of the trial might be biased towards the less technically challenging procedure.

In the trial by Brubaker and colleagues,[4] nothing was mentioned about the minimum number of procedures a surgeon must have done to be eligible to participate in the study; this is of minimal importance because both abdominal sacrocolpopexy and Burch colposuspension are common and well standardized procedures. Neither is a “new” technique, hence, the risk of differential expertise bias is low. However, an important point highlighted by the trial methodology was the fact that approximately one-third of the participating surgeons frequently performed paravaginal repair at the time of the sacrocolpopexy.[3] This was taken into consideration in the methods section since the groups were stratified according to the surgeon and intention to perform paravaginal repair, which was done at the surgeon's discretion and disclosed before randomization. This ensures equal proportion of surgeons frequently performing paravaginal repair in both groups.

### Were the patients randomized?

If prognostic factors, either known or unknown, prove to be unbalanced between the two treatment groups, the outcome will be biased, resulting in either an underestimation or overestimation of the treatment effect. Because patients’, physicians’, or outcome assessors’ preferences as a result of known prognostic factors may bias group allocation, randomization removes this potential bias by ensuring that both known and unknown prognostic factors in participating patients will be evenly balanced between the study groups[10] which is a prerequisite to begin a study.

It is important to critically assess the method of randomization. Of the available methods of randomization, a centrally-located, computer-generated randomization is the best method to minimize the risk of selection bias. Although some trials are labeled as randomized, they use inappropriate randomization methods, for example, odd or even birth year, day of the week, alternate chart number, the surgeon on call and so on. These trials should be labeled as “quasi-randomized.” The potential problem with these randomization techniques is selection bias.[11] For example, if the person responsible for recruiting patients into the trial knows beforehand, either because of the chart number or the day of the week, that the next eligible patient will be randomized to Group A, that person might elect, consciously or unconsciously, to not recruit the patient into the study for whatever logistic reasons, hence introducing a bias into the trial.

In the study by Brubaker and colleagues,[4] the authors clearly indicated their method of randomization by stating that they used “computer-generated random numbers in blocks of various sizes.” The use of blocks of various sizes means that at one round of randomization say two patients will be allocated to each group and at another round of randomization say four patients will be allocated to each group. This makes it difficult to predict which group the patient will be allocated to, hence, minimizing the risk of selection bias.

### Was the allocation concealed?

Concealment of allocation is the second safeguard after randomization to ensure that two groups begin a study with similar prognosis. As indicated earlier, if those making the decisions about patient eligibility are aware of the group to which patients will be allocated, either because of poor randomization technique or because the randomization is not concealed, they may systematically (either consciously or unconsciously) enroll patients with better prognosis into either the treatment or control group. There are different methods of allocation concealment. For example, in pharmaceutical trials, it is much easier to conceal allocation by preparing the medication in a blinded fashion in a pharmacy. On the other hand, in surgical trials, the most effective way of ensuring concealment is central randomization, in which the person recruiting patients calls an uninvolved party or methods center to discover to which group the patient is allocated. An alternative method of allocation concealment is the use of sequentially-numbered, sealed, opaque envelopes that contain the group allocation. The use of sequentially-numbered envelopes safeguards against repeated opening of envelopes and combination of them being sealed and opaque makes it very difficult to know group allocation without breaking the seal.

In the study by Brubaker and colleagues,[4] the authors declared the method of allocation concealment, stating that “assignment was revealed in the operating room (as information contained in sealed, opaque envelopes) after the woman was anesthetized.” Since the authors did not mention any means that was used to protect against repeated opening of their opaque envelopes, we cannot confirm that allocation concealment was optimum in this study.

### Were the patients stratified?

The third safeguard after randomization and allocation concealment to ensure similar prognosis of the two groups at the beginning of a study is stratified randomization. This entitles the even distribution of any prognostic factor known to be strongly associated with the outcome.

In surgical trials, it is impossible to blind surgeons, thus, they are sources of bias. Although the trigger for conducting the trial under question is usually clinical uncertainty or equipoise, surgeons usually have personal preferences to certain procedures either because of training background or technical demands. Thus, the surgeon might, consciously or subconsciously, bias the results of the procedure he believes in, by different methods:[6] 1) differential performance bias: spending more time and more attention on one procedure over the other; 2) differential co-intervention bias: higher chance of prescribing co-intervention, or conducting additional surgical procedure, to the group that received the procedure of preference; and 3) differential procedural crossover bias: the likelihood of the surgeon to crossover to the other procedure is higher in surgeons with less experience in the procedure to which their patient was assigned to. Therefore, stratification by surgeon should be considered whenever possible in surgical trials.

In the study by Brubaker and colleagues,[4] the authors indicated that “groups were stratified according to surgeon and intention to perform paravaginal repair (done at the surgeon's discretion and disclosed before randomization).” This ensures that the surgeons, who are potential sources of bias in this study, are evenly distributed among the two groups.

### Were all patients’ data analyzed in the group to which they were randomized?

The fourth safeguard to ensure that the known and unknown prognostic factors remain equally distributed between the two groups as they were at randomization is the principle of intention-to-treat analysis. Basically, intention-to-treat analysis means that patients’ data are analyzed within the group to which they were randomized, regardless of whether they received the assigned treatment or not.[9,12] Although it might not be intuitive to include such patients in the analysis if they did not receive the assigned treatment, the following discussion will clarify this concept. For example, if a patient was assigned to receive procedure A, but for medical reasons he or she became unstable intra-operatively, the surgeon will decide to choose whichever procedure that would take less time, regardless of the study protocol. Similarly, if the patient was assigned to receive the same procedure A, but because of technical difficulty (e.g. poor soft tissue quality, bleeding, and so on), the surgeon decides to do the alternative procedure (procedure B). In both examples, both patients are inherently different because of their prognostic factors (unknown at the time of randomization, but were declared during intervention), i.e. medical instability, and poor soft tissue quality. If these patients were dropped from their assigned group (A) and analyzed according to the treatment they received (B), then only good prognosis patients are included in Group A. This will result in overestimation of the treatment effect in Group A. In the study by Brubaker and colleagues,[4] the authors performed intention-to-treat analyses.

### Were the patients in the two groups similar with respect to known prognostic factors?

So far, we have assessed four measures that ensure that the two groups under investigation begin the study with similar prognosis for surgical randomized trials. A final step will be to assess how successful the four measures are in achieving balanced prognostic factors between the two groups. Although we cannot confirm whether unknown prognostic factors are distributed evenly between the two groups, the confirmation of equal distribution of known prognostic factors provide assurance of similar prognosis of the two groups at the beginning of the study. What we are interested in is not the mere statistical difference between the baseline characteristics (known prognostic factors are commonly presented in trials in the first table of the results section) but the magnitude of difference and the clinical significance of such difference between the two groups. The larger the magnitude of discrepancy between the known prognostic factors (baseline characteristics) and the stronger the association between the prognostic factors and the outcome, the more differences will weaken the validity of the results of the study and compromise the inference about the treatment effect.

If, despite all the safeguards implemented, the known prognostic factors (or baseline characteristics) are clinically significantly different between the two groups, statistical techniques can be used to adjust the results for the differences in the baseline characteristics. Hence, it is important to compare both the adjusted and unadjusted analyses. If both lead to the same conclusion, only then we can confidently believe that the results of the study are valid.

In the study by Brubaker and colleagues,[4] the authors assured us that there were no differences in the baseline characteristics between the two groups. This is confirmed by our own review of the baseline characteristics of the patients in both groups. However, since the randomization was stratified according to surgeon and intention to perform paravaginal repair, the analysis was adjusted for surgeon and the presence or absence of paravaginal repair.

#### 2. Did the two groups retain a similar prognosis after the study started?
Were the patients, surgeons and outcome assessors aware of group allocation?

In other words, were the patients, surgeons and outcome assessors blinded to the group allocation? It is better to explicitly state who was blinded in the trial, rather than using the terminology double- or triple-blinded because of the confusion about this terminology.[13]

*Were the patients blinded?* It has been documented in the medical literature that if patients know their treatment allocation and believe that it is more beneficial (especially if it is a “new” procedure); they tend to feel better than patients who do not, even if they received the same treatment.[14] This is termed the placebo effect.[9] This is of more significance if the outcome of interest is subjective, such as patient-reported quality of life measures. Intuitively, if patients feel better because they know that they received the “new” treatment, they will answer the quality of life questions more favorably biasing the results in favor of the “new” treatment. The placebo effect is of less importance if the outcome of interest is a relative objective outcome, for example, development of a wound infection, deep venous thrombosis or death. Every effort should be made by the investigators to decrease the risk that the patients become aware of their treatment allocation, for example, using similar incisions, or similar wound dressing to mask the underlying differences in the procedure. If the trial compares operative versus non-operative treatment, it is impossible to blind the patients to their group allocation. Although sham surgery is a potential solution, the ethical complexity of this solution prevents its widespread usage.[15,16] In such situations where it is impossible to blind patients, it is very important to choose an objective outcome to decrease the bias of the placebo effect.

*Were the surgeons blinded?* It is impossible to blind surgeons. Thus, the surgeon might, consciously or subconsciously, bias the results of the procedure he believes in, by different methods[6] as mentioned earlier: 1) differential performance bias, 2) differential co-intervention bias, and 3) differential procedural crossovers bias. Apart from stratification, this problem has no easy solution. Devereaux and colleagues proposed the use of expertise-based randomized trial design as a potential solution to this problem.[6] In this design, patients are randomized to different surgeons with expertise in the relevant intervention. They showed how investigators have used expertise-based design when conventional trial design was impossible because different specialty groups provided the interventions under investigation, for example percutaneous transluminal coronary angioplasty versus coronary artery bypass graft surgery.[17–19] According to Devereaux and colleagues,[6] the advantage of the expertise-based randomized controlled trial is that surgeons will perform only the procedure in which they have expertise, avoiding the problem of differential expertise. Although surgeons in the expertise-based randomized controlled trial will still be unblinded, they are likely to be subconsciously biased toward the procedure in which they have expertise. Consequently, the likelihood of differential procedural performance, co-interventions, and procedural crossovers are less likely to occur because surgeons are doing the procedures with which they are most comfortable.

*Were the outcome assessors blinded?* Outcome assessors are those who collect outcome information, whether it is the surgeons themselves or more preferably independent research nurses. If the outcome assessors are unblinded, they may provide different interpretations of marginal results or differential degree of encouragement during performance tests, either of which can bias the results.[20] Similarly, if one group receives close follow-up, outcomes may be reported more frequently in that group thus biasing the results of the study. Outcome assessors can almost always be blinded even if the patients and surgeons cannot. An additional safeguard against outcome assessment bias commonly used in large randomized trials is the assembly of a blinded adjudication committee to review the patients’ data and decide whether a patient has an outcome.

In the study by Brubaker and colleagues,[4] the authors indicated that the patients, research staff, and telephone interviewers were unaware of the treatment assignment for a minimum of three months and blinding was intended to be maintained for two years after surgery. In the results section, they highlighted that the group allocation was revealed (unblinded) to study coordinators before the three-month visit in the case of five women and to the women themselves in two cases. The surgeons were obviously unblinded; however, randomization was stratified by surgeon to decrease the risk of differential experience bias. The trial was a conventional randomized trial design, that is, the patients were randomized to receive either the control treatment (abdominal sacrocolpopexy) or the intervention treatment (abdominal sacrocolpopexy and Burch colposuspension). This was not an expertise-based randomized trial, i.e., patients were not randomized to be treated by either a surgeon who usually performs the control treatment or a surgeon who usually performs the intervention treatment.

### Was the follow-up complete?

The failure to account for all the patients at the end of a study is a major threat to a study's validity. Patients whose status is unknown are referred to as having been lost to follow-up. The greater the number of patients lost to follow-up, the greater the harm done to the study's validity. The reason is that patients who are lost to follow-up are usually systematically different in terms of their prognoses compared with the rest of the patients who were compliant with the follow-up regimen. Patients who are lost to follow-up have either died or have had an adverse outcome and might have sought another medical advice or doing very well and thus did not bother to return for follow-up. Although there is no magic number for the cutoff point for an acceptable loss of follow-up rate, there is some sort of agreement that if more than 20% of patients are lost to follow-up, the validity of the study is questionable.

Since the loss of follow-up is beyond the control of the investigators, there are solutions to this problem. First, anticipating a loss of follow-up based on the population in the study and taking that into consideration when calculating the sample size of the study. Second, conduct best-case scenario and worse-case scenario analyses of patients' data. Best-case scenario analysis means conducting analysis assuming that all patients lost to follow-up have a good outcome, while worst-case scenario analysis means assuming that all patients lost to follow-up have a bad outcome. If both analyses reach the same conclusion, then the loss of follow-up is of minimal influence on the validity of the results.

In the study by Brubaker and colleagues,[4] the primary outcomes of the study were stress incontinence and urge symptoms three months after surgery. Although there was no loss to follow-up at the three-month follow-up visit, the stress incontinence end point could not be determined for 10 women in the Burch group (6%) and 13 in the control group (8%). The authors conducted best- and worst-case scenario intention-to-treat analyses and found statistically significant differences between the two groups in favor of the Burch group when these analyses were used.

The previous sections summarized the first part of the critical appraisal to answer an important question: Are the results of the study by Brubaker and colleagues[4] valid? We have answered this question in detail within each section; however, it is a good idea to summarize the answer before embarking into answering the second question: What are the results? The study by Brubaker and colleagues[4] comparing abdominal sacrocolpopexy alone with abdominal sacrocolpopexy combined with Burch colposuspension was in fact of high methodological quality and, therefore, should yield valid results. They used computer-generated random numbers in blocks of various sizes. Their allocation concealment technique was the use of sealed, opaque envelopes, although we are not sure whether the envelopes were sequentially numbered or not. The groups were stratified according to surgeon and intention to perform paravaginal repair. The authors performed intention-to-treat analyses with additional analyses adjusted for surgeon and the presence or absence of paravaginal repair. The authors indicated that there were no differences in the baseline characteristics between the two groups, which our own review confirmed. Patients, research staff, and telephone interviewers were unaware of the treatment assignment for a minimum of three months (except for the study coordinators in five occasions and the patients for two occasions). The surgeons were not blinded; however, randomization was stratified by surgeon to decrease the risk of differential experience bias. Finally, although there was no loss of follow-up at the three-month follow-up visit, the primary outcome (stress incontinence) was not available for 6% of the patients in the Burch group and 8% in the control group. The authors conducted best- and worst-case scenario analyses and continued to find significant differences in favor of the sacrocolpopexy group, thereby supporting their study findings.

## CONCLUSION

The assessment of methodological quality which entails a close review of the material and methods of a given study should always stand at the beginning of every critical appraisal. While randomized controlled trials have the potential of providing the highest quality of evidence, important methodological shortcomings can draw into question the confidence we can play in the results. It is therefore of critical importance that the key methodological characteristics that represent safeguards against bias are reported in the literature as suggested by the Consolidated Standards of Reporting Trials (CONSORT) criteria. Meanwhile, a recent study of the urological literature suggests that reporting quality in major urological journals is low, thereby hindering the critical appraisal process.[21] Unfortunately, very few urological journals have yet to formally endorse the CONSORT criteria for the transparent reporting of RCTs, which has been shown to improve reporting quality.[22]

Once the readers have determined that the results of a study are likely valid, it is important to review the actual results and determine whether they can and should be applied to the care of an individual patient. These are the recommended second and third steps in a three-tiered critical appraisal process that will be detailed in the second part of this review on evidence-based urology.
